# New COPE guidelines on publication process manipulation: why they matter

**DOI:** 10.1186/s41073-018-0059-x

**Published:** 2018-11-26

**Authors:** Jigisha Patel

**Affiliations:** Springer Nature, London, UK

## Abstract

Manipulation of the publication process is a relatively new form of misconduct affecting the publishing industry. This editorial describes what it is, why it is difficult for individual journal editors and publishers to handle and the background to the development of the new COPE guidelines on how to manage publication process manipulation.

These new guidelines represent an important first step towards encouraging openness and collaboration between publishers to address this phenomenon.

10 years ago, a retraction of an article was a rare thing. We know that the rate of journal retractions has been rising [1]. It has been argued that the increasing number of journals and the ‘pressure to publish’ have been the driving unethical practices such as data falsification, fabrication, and plagiarism [2]. There have been calls to address this by changing the way research success is measured, for example, by changing the way journal and article quality is measured and rewarded [3] in the hope that, by removing the pressure, unethical practices might decline.

About 4 years ago, a new problem began to emerge. Even those of us who are used to seeing all sorts of misconduct related to research were surprised. The problem was not due to the actions of individual researchers behaving unethically on individual publications, but something very different, systematic, and organized. The problem was of researchers or third parties systematically manipulating publication processes to either boost their own publication records or to exploit the ‘publish or perish’ culture of scientific research for profit. The process involved manipulating the publication process to ensure articles passed peer review and, in some cases, collecting a fee from the authors for this service, or offering authorship of the article for a fee. A number of publishers (including the publisher of this journal) have experienced some form of systematic manipulation of their publication processes and have retracted the affected articles (for example, [4–7]).

Publication process manipulation is an industry-wide problem. The full extent of it is yet unknown and many publishers will have little experience of how to identify and manage it. For individual journals and editors, it is extremely difficult to spot. Even if detected, the competitive environment of commercial publishing together with confidentiality issues naturally discourage individual publishers from disclosing their methods of investigation, findings, and measures taken to prevent manipulation of their systems. However, to effectively tackle publication manipulation, a coordinated and collaborative approach is needed. With this thinking in mind, members of the Springer Nature Research Integrity Group [8], in collaboration with the Committee on Publication Ethics (COPE) [9], ran a workshop on publication manipulation at a COPE Publisher’s seminar in October 2016. At the seminar, a group of publishers were brought together to share their experiences of publication manipulation practices. The ultimate aim of the workshop was to agree a standardized approach to investigating and resolving such issues.

Work has continued since the workshop and, after much collaboration and many iterations, COPE now has new guidelines and flowcharts on publication manipulation [10].

These guidelines provide advice on how to spot and investigate publication manipulation, when to involve institutions, and what actions to take. As well as a valuable addition to COPE’s extensive existing collection of resources, these guidelines are important because, by acknowledging that there is an industry-wide problem of publication manipulation, they represent a first step towards encouraging openness and collaboration between publishers to address this phenomenon.
